# RANTES/CCL5 Induces Collagen Degradation by Activating MMP-1 and MMP-13 Expression in Human Rheumatoid Arthritis Synovial Fibroblasts

**DOI:** 10.3389/fimmu.2017.01341

**Published:** 2017-10-18

**Authors:** Solomon A. Agere, Nahid Akhtar, Jeffery M. Watson, Salahuddin Ahmed

**Affiliations:** ^1^Department of Pharmaceutical Sciences, College of Pharmacy, Washington State University, Spokane, WA, United States; ^2^Department of Chemistry and Biochemistry, Gonzaga University, Spokane, WA, United States; ^3^Division of Rheumatology, University of Washington School of Medicine, Seattle, WA, United States

**Keywords:** rheumatoid arthritis, synovial fibroblasts, regulated on activation, normal T expressed, secreted/CC ligand 5, matrix metalloproteinases, heparan sulfate proteoglycans

## Abstract

Regulated on activation, normal T expressed, and secreted (RANTES)/CC ligand 5 (CCL5) participates in rheumatoid arthritis (RA) pathogenesis by facilitating leukocyte infiltration, however, its other pathological functions are not fully defined in RA. In the present study, we evaluated the effect of RANTES/CCL5 on tissue degrading enzymes matrix metalloproteinase-1 (MMP-1) and MMP-13 expression and its contribution to the progressive joint damage by RA synovial fibroblasts (RASFs). Our results showed that RANTES/CCL5 dose dependently induced MMP-1 and MMP-13 expression in monolayers and three-dimensional (3D) micromass of human RASFs, which correlated with an increase in collagenase activity. This activation by RANTES/CCL5 was observed in RASF, but not in osteoarthritis SFs (OASFs). Evaluation of the signaling events showed that RANTES/CCL5 selectively activated PKCδ, JNK, and ERK proteins to induce MMP expression in human RASFs. Pretreatment with a functional antagonist (Met-RANTES) or heparinase III [an enzyme that selectively digests heparan sulfate proteoglycans (HSPGs)] completely abrogated RANTES/CCL5-induced MMP-1 and MMP-13 expression. Interestingly, the inhibition of RANTES/CCL5 using small-interfering RNA approach reduced the ability of interleukin-1β (IL-1β) to induce MMP-1 and MMP-13 expression, asserting its mediatory role in tissue remodeling. In the inhibitor study, only the selective inhibition of HSPGs or PKCδ, ERK, and JNK markedly inhibited RANTES/CCL5-induced MMP-1 and MMP-13 production. Circular dichroism spectroscopy results demonstrated the degradation of collagen triple-helical structure upon exposure to the conditioned media from RANTES/CCL5 stimulated RASFs, which was reverted by a broad-spectrum MMP inhibitor (GM6001). These findings suggest that RANTES/CCL5 not only upregulates MMP-1 and MMP-13 expression by partly utilizing HSPGs and/or PKCδ-JNK/ERK pathways but also mediates IL-1β-induced MMP-1 and MMP-13 expression.

## Introduction

Rheumatoid arthritis (RA) is an autoimmune disease in which activated synovial fibroblasts (SFs) produce chemokines to facilitate infiltration of inflammatory cells (1–4). Chemokines are classified in to four subfamilies (C, CC, CXC, and CX_3_C) depending on the number and spacing of cysteine motif (1, 5). Chemokines exert their effect through the recruitment and retention of monocytes and T lymphocytes in the joints, leading to hyperplasia of the synovial lining, and causing the destruction of bone and articular cartilage (4, 6, 7). In response to interleukin-1β (IL-1β) and tumor necrosis factor α (TNF-α), RA synovial fibroblasts (RASFs) release chemokines that bind to their receptors to recruit inflammatory cells at the site of inflammation (8). Among these chemokines, regulated on activation, normal T expressed and secreted (RANTES)/CC ligand 5 (CCL5) is a potent CC chemokine shown to play an important role in RA pathogenesis (9); however, its role beyond chemotactic activity is not well defined in RA. RANTES/CCL5 primarily attracts lymphocytes and monocytes as well as other cell types (1, 2). RASFs produce RANTES/CCL5 upon stimulation with TNF-α, IL-1β, or interferon gamma (10).

Matrix metalloproteinases (MMPs) are matrix degrading enzymes that have high affinity to destroy or remodel extracellular matrix (ECM) (1, 11, 12). Among different MMPs, the collagenases are of particular importance as they are able to efficiently cleave collagen. MMP-1 and MMP-13 are collagenases that play a significant role in bone and cartilage degradation (13). In RA, the transformation of normal synovial lining into a hyperplastic, invasive tissue pannus utilizes MMP-1 and MMP-13 to erode joint tissue (11, 14). In particular, MMP-1 and MMP-13 are capable of degrading intact fibrillary collagen that provides strength to cartilage (11, 15).

In the present study, we evaluated the effect of RANTES/CCL5 in inducing MMP-1 and MMP-13 expression in human RASFs and its underlying mechanism of action. Furthermore, we examined the effect of RANTES/CCL5-induced MMPs on the structure of native collagen.

## Materials and Methods

### Reagents and Antibodies

Recombinant human RANTES/CCL5, Met-RANTES and IL-1β, and MMP-1 ELISA Duoset were purchased from R&D systems (Minneapolis, MN, USA). MMP-13 ELISA was purchased from Ray Biotech (Norcross, GA, USA). MMP-1 and MMP-13 SYBR Green primers were purchased from Qaigen (Valencia, CA, USA). SMARTpool ON-TARGETplus RANTES small-interfering RNA (siRNA) and On-target plus non targeting siRNA (D-001810-10) control were purchased from GE Dharmacon (Lafayette, CO, USA). MMP-1, MMP-13, type I collagen, and lamin A/C antibodies were purchased from Santa Cruz Biotech (Santa Cruz, CA, USA). Rabbit monoclonal or polyclonal antibodies against p-p38 (Thr^180^/Tyr^182^), p-ERK (Thr^202^/Tyr^204^), p-JNK(Thr^183^/Tyr^185^), p-PKCδ (Thr^505^), total p-38, total JNK, total ERK, p-c-Jun (Ser^73^), and p-ATF-2 (Thr^69/71^) were from Cell Signaling Technologies (Beverly, MA, USA). Rabbit polyclonal antibodies against CCR1 and CCR5 were from BioVision (Milpitas, CA) and GeneTex (Irvine, CA, USA), respectively. Heparinase III (flavobacterium heparinum derived), heparan sulfate degrading lyase was purchased from Sigma (St. Louis, MO, USA). Type I collagen was purchased from Advanced Biometrix (Carlsbad, CA, USA). Inhibitors of JNK (SP600125), ERK (PD98059), p38 (SB 02190), PKCδ (Rottlerin), and NF-κB (PDTC) were purchased from EMD Millipore (Billerica, MA, USA).

### Culture of Human RASFs

Deidentified RA synovial tissues were procured under a protocol approved by the Washington State University IRB (IRB#14696) from patients with diagnosed RA or OA who had undergone total joint replacement surgery or synovectomy and in compliance with the Declaration of Helsinki. No consent was needed because investigators had access only to deidentified human materials that were not collected specifically for the purpose of this research and for which they do not have ready means to link back to living individuals. The deidentified human normal (NL), RA, or OA synovial tissues were obtained from the Cooperative Human Tissue Network (Columbus, OH, USA) and the National Disease Research Interchange (Philadelphia, PA, USA). Some RASF patient cell lines were provided by our rheumatologist collaborator Dr. David A. Fox (University of Michigan, Ann Arbor, USA). RASFs were isolated through enzymatic digestion and grown in RPMI 1640 containing 2 mM l-glutamine with 10% FBS growth medium at 37°C in a humidified atmosphere with 5% CO_2_ as described earlier (16, 17). RASFs between passages 5 and 10 were used in the experiments. All treatments were performed in serum-free medium.

### Treatment of RASFs

To evaluate the effect of RANTES/CCL5 on MMP-1 and MMP-13 expression, human NLSFs, RASFs or OASFs were treated with recombinant human RANTES/CCL5 (20, 50, or 100 ng/ml) for 24 h. Human RASFs were also pretreated with Met-RANTES (a functional antagonist of RANTES; 50, 100, and 200 ng/ml) for 30 min and then stimulated with RANTES/CCL5 or IL-1β for 24 h. Total RNA and conditioned media was used to study the MMP-1 and MMP-13 expression using qRT-PCR, ELISA, and Western immunoblotting, respectively. Effect of RANTES/CCL5 on the activation of NF-κB, MAPK, and PKCδ signaling pathways was determined in RASF lysates treated with RANTES/CCL5 (100 ng/ml) for 5, 15, and 30 min using Western immunoblotting. For some experiments, RASFs were pretreated with the inhibitor of p38 (SB203980; 10 µM), ERK (PD98059; 10 µM), JNK (SP600125; 10 µM), NF-KB (PDTC; 200 µM), or PKCδ (Rottlerin; 10 µM) for 2 h followed by RANTES/CCL5 treatment for 24 h. Culture supernatants were concentrated using Amicon^®^ Ultra centrifugal filters (Millipore) and MMP-1 and MMP-13 expression was determined using Western immunoblotting.

To study the effect of Met-RANTES on IL-1β-induced signaling pathways, we pretreated RASFs with Met-RANTES (100 ng/ml) for 30 min, followed by IL-1β (10 ng/ml) stimulation for 30 min. Cell lysates were prepared to be used for the analysis of the phosphorylated proteins (p-PKCδ, p-JNK, and p-ERK).

To study the role of heparan sulfate proteoglycans (HSPGs) in RANTES/CCL5-induced MMP production, RASFs were pretreated with heparinase III (0.5 U/ml), an enzyme that recognizes HSPGs as its primary substrate, for 2 h and then stimulated with RANTES/CCL5 (100 ng/ml) for 24 h. Conditioned media was concentrated to determine MMP-1 and MMP-13 expression by Western immunoblotting.

To verify whether heparinase III affects the binding of RANTES to its receptor to inhibit the biological activity, we pretreated RASFs with heparinase III for 2 h and then stimulated with RANTES/CCL5 (100 ng/ml) for 1 h. We ran this experiment in two sets both in triplicate. In the first set, we terminated the reaction without cross-linking proteins by washing with ice-cold PBS three times. In the second set, we cross-linked proteins with 1% formaldehyde for 10 min followed by 125 mM glycine neutralization for 5 min, and then washed three times with cold PBS. Whole cell lysates were prepared using RIPA buffer and an equal amount of protein in 25 µl was used for detecting bound RANTES using a commercially available kit (R&D Systems). RANTES/CCL5 levels were normalized with per mg of cellular protein.

### Human RASFs Three-Dimensional (3D) Micromass Cultures

Micromass organ cultures were constructed as described by Kiener et al. (18). Briefly, RASFs were released from the culture dish using 0.02% (weight/volume) TPCK-treated trypsin (Worthington, Lakewood, NJ, USA) in HEPES buffered saline solution (20 mM HEPES, 137 mM NaCl, and 3 mM KCl, pH 7.4) containing 2 mM CaCl_2_. Cells were resuspended in ice-cold Matrigel Matrix (BD Biosciences) at a density of 5 × 10^6^ cells/ml. Droplets of the cell suspension (25 µl) were placed onto 12-well culture ultralow attachment dishes (Corning, NY, USA). Thereafter, RASFs were cultured in basal culture medium (DMEM supplemented with penicillin, streptomycin, l-glutamine, nonessential amino acid solution, insulin–transferrin–selenium (BioWhittaker, Rockland, ME, USA), 0.1 mM ascorbic acid, and 10% heat-inactivated FBS). The floating 3D culture was maintained for 3 weeks; the medium was routinely replaced twice weekly. For experiments, RASFs were pretreated with Met-RANTES (200 µg/ml) for 30 min followed by stimulation with RANTES/CCL5 (100 ng/ml) for 24 h. IL-1β (10 ng/ml) for 24 h was used as a positive control. Culture supernatant were concentrated using Amicon^®^ Ultra Centrifugal filters (Millipore) and MMP-1 and MMP-13 expression was determined using Western immunoblotting.

### Transient Transfection of siRNA

To study the effect of RANTES/CCL5 knockdown on MMP-1 and MMP-13 production, RASFs were transfected with ON-TARGET plus SMART pool RANTES/CCL5 siRNA (GE Dharmacon, Lafayette, CO, USA) using Lipofectamine^®^ RNAiMAX (Life Technologies) for 48 h and then stimulated with IL-1β (5 ng/ml) for 24 h. Conditioned media was used to study the effect of MMP-1 and MMP-13 production using ELISA.

### Western Immunoblot Analyses

Western blot analysis on the cell lysates and conditioned media was performed as described in our earlier studies (16, 19). Images were analyzed using the GELDOC or Image-J software. Each band was scanned using Image lab 5.1 software and the expression values (pixels/band) were presented as mean ± SE.

### In-Gel Zymography

Collagenase activity in the RANTES/CCL5-treated conditioned media was determined by the in-gel zymography method as described earlier (19). Fifteen microliter of the conditioned media was resolved under non-reducing conditions on SDS-polyacrylamide gels loaded with collagen (1 mg/ml: type A from porcine skin; Sigma, St. Louis, MO, USA) as a substrate. Following electrophoresis, the gels were washed with 2.5% Triton X-100 for 30 min with gentle shaking, followed by a 30 min or overnight wash in developing buffer (50 mM Tris–HCl, pH 8.0, 5 mM CaCl_2_, and 0.02% NaN_3_). Finally, gels were stained in Coomassie blue (R-250) and images of the digested regions representing MMPs activity were captured and analyzed using Image-J software.

### Quantitative Real-time PCR Analysis

Total RNA was reverse-transcribed using SuperScript™ first Strand synthesis kit (Life Technologies, Carlsbad, CA, USA) according to the manufacturer instruction. The mRNA expression using validated primers for MMP-1 (NM_002421; Cat. No. QT00014581, Qiagen) and MMP-13 (NM_002427; Cat. No. QT00001764, Qiagen) was quantified using the power SYBR^®^ Green PCR master mix (Life Technologies) and QuantiTect primer assay (Qiagen). GAPDH mRNA expression was used as endogenous control. Quantification of the relative expression was determined by ΔΔCt method.

### Preparation of Nuclear Extracts

To study activation and nuclear translocation of p-c-Jun and p-ATF-2, RASFs were treated with RANTES/CCL5 (100 ng/ml) for 5, 15, and 30 min. Nuclear fractions were prepared as described before (1) and evaluated for p-c-Jun and p-ATF2 expression using Western immunoblotting.

### *In Vitro* JNK/SAPK Kinase Assay

Rheumatoid arthritis synovial fibroblasts were pretreated with Met-RANTES for 30 min and stimulated with or without RANTES/CCL5 (100 ng/ml) for 30 min. Cells were washed twice with ice-cold PBS and scrapped directly into 0.25 ml of lysis buffer provided with the kinase assay kit. *In vitro* JNK/SAPK kinase assay was performed using a non-radioactive kinase assay kit according to the instruction of the manufacturer (Cell Signaling Technology) as described earlier (12).

### Collagen Degradation Assay

Human RASFs were pretreated with or without GM6001 (a broad-spectrum MMP inhibitor; 20 µM) for 2 h and then stimulated with RANTES/CCL5 (100 ng/ml) or IL-1β (10 ng/ml). A modified collagen degradation assay method was followed (20) in which the conditioned media from the above experiments were added onto a 96-well plate coated with type I collagen (200 μg/well) and incubated for 24 h at 37°C. After removing the condition media, the plates were stained with Coomassie R250, washed to remove soluble stain, and analyzed by ChemiDoc™ XRS Imager (Bio-Rad). In addition, the conditioned media (500 µl) collected after treating type I collagen was concentrated using Amicon 10K ultra 0.5 ml centrifugal filters and resolved on 7.5% SDS-PAGE using for COL1A1 detection by immunoblotting.

### Circular Dichroism (CD) Spectroscopy

Condition media from RANTES/CCL5 or IL-1β stimulated RASFs was applied on collagen-coated 96-well plates in triplicate as described above for 8 and 48 h. Condition media was removed and plates were carefully washed with PBS to remove media residue. Remaining digested collagen was collected using 200 µl of 0.1% acetic acid. CD spectroscopy was performed using a Jasco J-815 spectropolarimeter outfitted with a Peltier temperature-controlled cell holder. All spectra were collected at 25°C in a 1 mm cuvette. CD signal was monitored between 200 and 250 nm wavelengths (21). Samples were normalized with 0.1% acetic acid as a blank. Native type I collagen was used as standard to compare the damage. We also performed a separate experiment to test whether there is any difference in the CD spectra when collagen is exposed to the conditioned media from untreated NLSFs and RASFs.

### Statistical Analysis

Statistical analysis was performed for the protein and mRNA *in vitro* data using one-way analysis of variance test, followed by Dunnett’s multiple comparison *post hoc* test with *p* < 0.05 chosen as the level of significance.

## Results

### RANTES/CCL5 Dose Dependently Induces MMP-1 and MMP-13 Expression in Human RASFs

**Figure 1:**
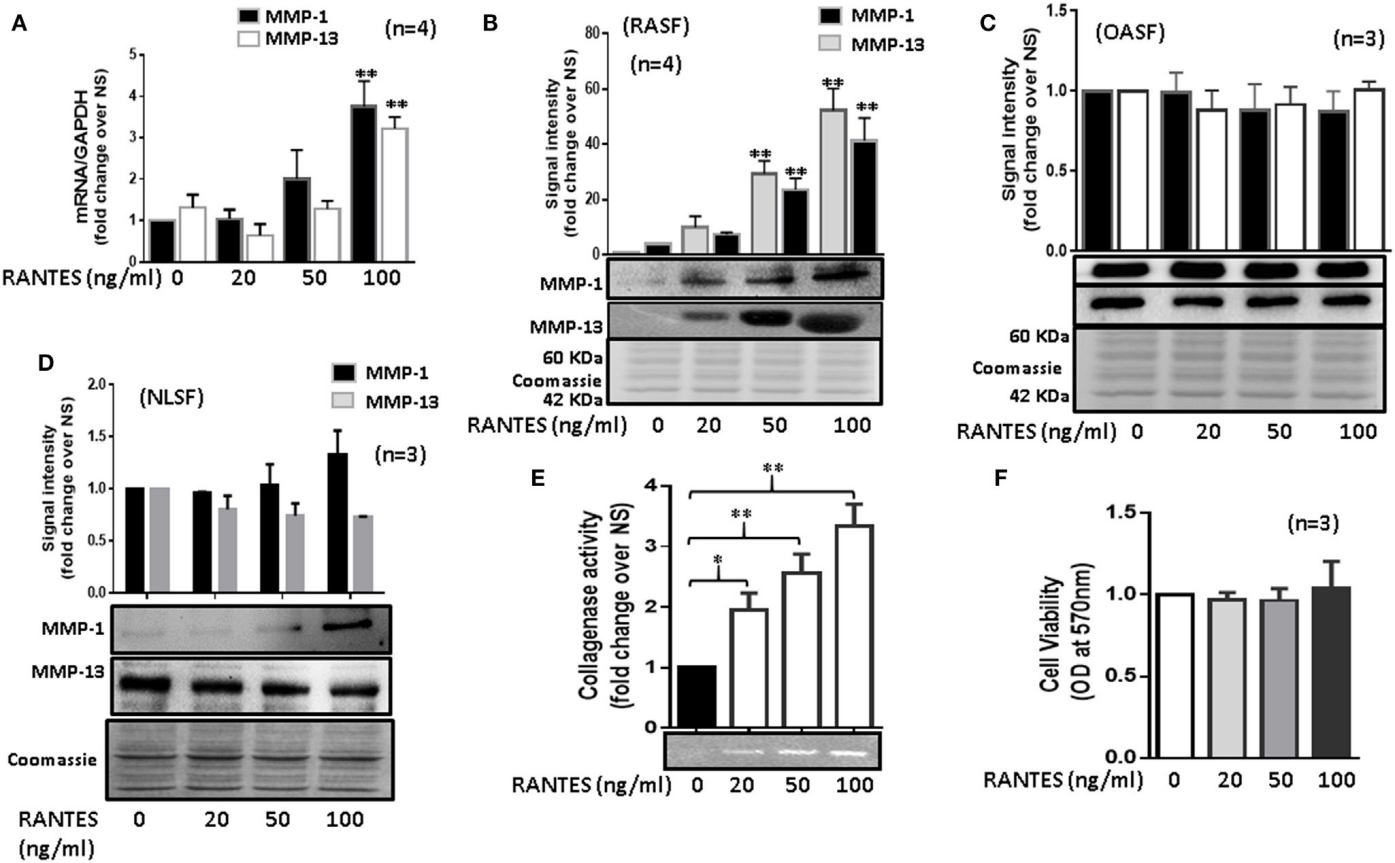
Regulated on activation, normal T expressed, and secreted (RANTES)/CC ligand 5 (CCL5) induces matrix metalloproteinase (MMP)-1 and MMP-13 expression in rheumatoid arthritis synovial fibroblasts (RASFs). **(A)** Effect of RANTES/CCL5 on MMP-1 and MMP-13 mRNA and **(B)** protein expression was studied in RASFs. **(C,D)** Human OASFs and NLSFs were treated with different concentrations of recombinant RANTES/CCL5 (20, 50, and 100 ng/ml) and MMP-1 and MMP-13 expression was analyzed using Western immunoblotting, respectively. Gels described for **(B–D)** were stained with Commassie Blue stain to ensure equal loading of the supernatants. **(E)** Effect of RANTES/CCL5 on collagenase activity in RASFs. RASFs were treated with different concentration (20, 50, and 100 ng/ml) of RANTES/CCL5 for 24 h and conditioned media was concentrated using Amicon^®^ Ultra Centrifugal filters (Millipore) and resolved on polyacrylamide gel loaded with collagen. Zymography image of digested regions representing MMPs activity is shown. Fold changes on developed zymograms was determined using densitometric analysis. **(F)** Effect of RANTES/CCL5 on the cell viability of RASFs was determined using MTT assay. RASFs were treated with RANTES/CCL5 (20, 50, and 100 ng/ml) for 24 h. Cell viability of cultured RASFs in the presence of RANTES/CCL5 was measured by optical densities at 570 nm. Values are represented as mean ± SE from three to four independent experiments using cells from different donors under similar conditions. *p < 0.05; **p < 0.01 for NS versus RANTES/CCL5 treatment.

Human RASFs treated with RANTES/CCL5 (20, 50, and 100 ng/ml) for 24 h showed a significant increase in MMP-1 (~2.3-fold) and MMP-13 (~2.1-fold) mRNA expression when compared to the untreated control (Figure 1A). A similar dose-dependent increase in the MMP-1 and MMP-13 protein production was observed upon RANTES/CCL5 stimulation (Figure 1B). Interestingly, RANTES/CCL5 had no inducing effect on MMP-1 and MMP-13 expression in OASFs (Figure 1C) or in the human dermal fibroblasts (data now shown), suggesting that RANTES/CCL5 preferentially inflicts tissue damage in RA. Surprisingly, RANTES/CCL5 marginally induced MMP-1 production, but dose-dependently reduced MMP-13 production in NLSFs (Figure 1D). To further validate that RANTES/CCL5-induced MMP-1 and MMP-13 expression in not due to differences in the expression of its receptor CCR5, and also CCR1, we performed Western blot analysis on the whole cell lysates prepared from untreated NLSFs and RASFs. Our results showed expression levels of CCR1 in RASFs was ~20% lower than NLSFs, however, there was no significant difference observed in the expression levels of CCR5 (Figure S1 in Supplementary Material).

To further verify the effect of RANTES/CCL5 on the enzymatic activity in RASFs, collagenase activity in RANTES/CCL5-stimulated conditioned media was determined using in-gel zymography. Densitometric analysis of the developed zymograms showed that RANTES/CCL5 dose dependently induced collagenase activity in RASFs (Figure 1E), without affecting RASF’s viability (Figure 1F). These findings suggest that RANTES/CCL5 selectively activates RASFs to produce MMP-1 and MMP-13 expression.

### Met-RANTES Inhibits RANTES/CCL5-Induced MMP-1 and MMP-13 Expression by RASFs

**Figure 2:**
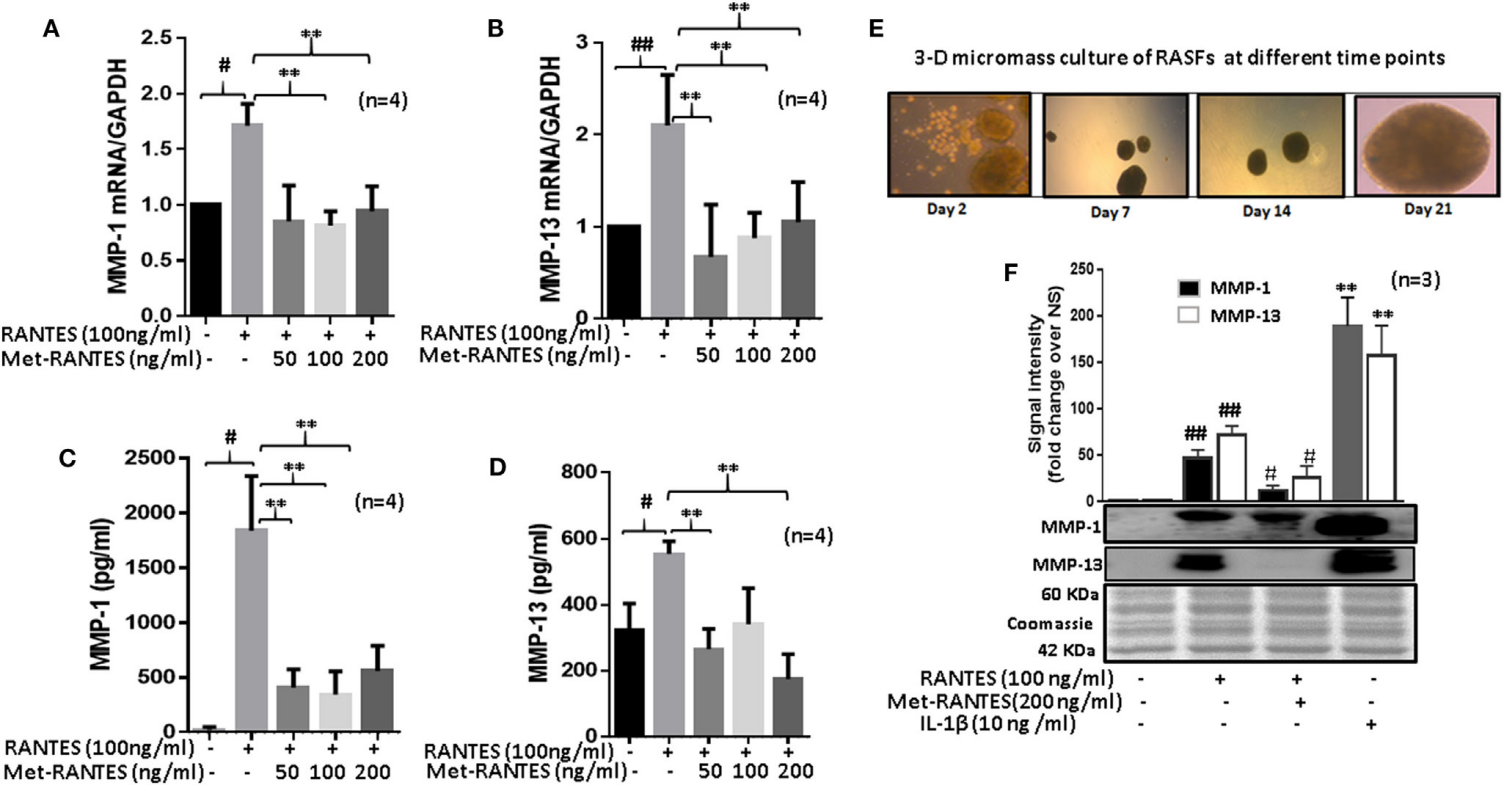
Met-RANTES inhibits regulated on activation, normal T expressed, and secreted (RANTES)/CC ligand 5 (CCL5)-induced matrix metalloproteinase (MMP)-1 and MMP-13 expression in rheumatoid arthritis synovial fibroblasts (RASFs). **(A,B)** Effect of Met-RANTES on RANTES/CCL5-induced MMP-1 and MMP-13 mRNA and **(C,D)** at protein levels was determined using qRT-PCR and ELISA, respectively. RASFs were pretreated with Met-RANTES (50, 100, and 200 ng/ml) for 30 min followed by stimulation with RANTES/CCL5 (100 ng/ml) for 24 h. **(E,F)** Human RASFs were cultured in three-dimensional (3D) micromass for 21 days to form synovial tissue-like architect, followed by pretreatment with or without Met-RANTES for 30 min and then stimulation with RANTES (100 ng/ml) for 24 h. Interleukin (IL)-1β (10 ng/ml) 24 h stimulated RASF micromass were used as the positive control. Conditioned media was concentrated using Amicon^®^ Ultra Centrifugal filters (Millipore) and MMP-1 and MMP-13 production was analyzed using Western immunoblotting. Densitometry was performed to determine the relative changes. Values are represented as mean ± SE from three to four independent experiments using cells from different donors. #*p* < 0.05 or ##*p* < 0.01 for NS vs. RANTES/CCL5 or IL-1β; **p < 0.01 for RANTES/CCL5 vs. Met-RANTES treatment. Representative figures of RASFs on 3D micromass culture (4× magnification; size 200–400 µm) are shown on days 2, 7, 14, and 21.

To study the effect of Met-RANTES on RANTES/CCL5-induced MMP-1 and MMP-13 production, human RASFs were pretreated with Met-RANTES (50, 100, and 200 ng/ml) for 30 min prior to RANTES/CCL5 stimulation for 24 h (Figure 2). MMP-1 and MMP-13 expression was determined using qRT-PCR and ELISA or Western immunoblotting. Met-RANTES significantly reduced RANTES/CCL5-induced MMP-1 (~53%) and MMP-13 (~66%) mRNA expression (Figures 2A,B). Analysis of the conditioned media from the same treatment showed a similar decrease in MMP-1 and MMP-13 production by Met-RANTES (Figures 2C,D). We also developed the 3D micromass culture of RASFs (Figure 2E) to verify the effect of Met-RANTES on RANTES/CCL5-induced MMP-1 and MMP-13 production in a synovial tissue-like environment, which showed a similar significant reduction of MMP-1 and MMP-13 in the condition media obtained from human RASFs micromass cultures (Figure 2F).

### RANTES/CCL5 Mediates IL-1β-Induced MMP-1 and MMP-13 Expression in RASFs

**Figure 3:**
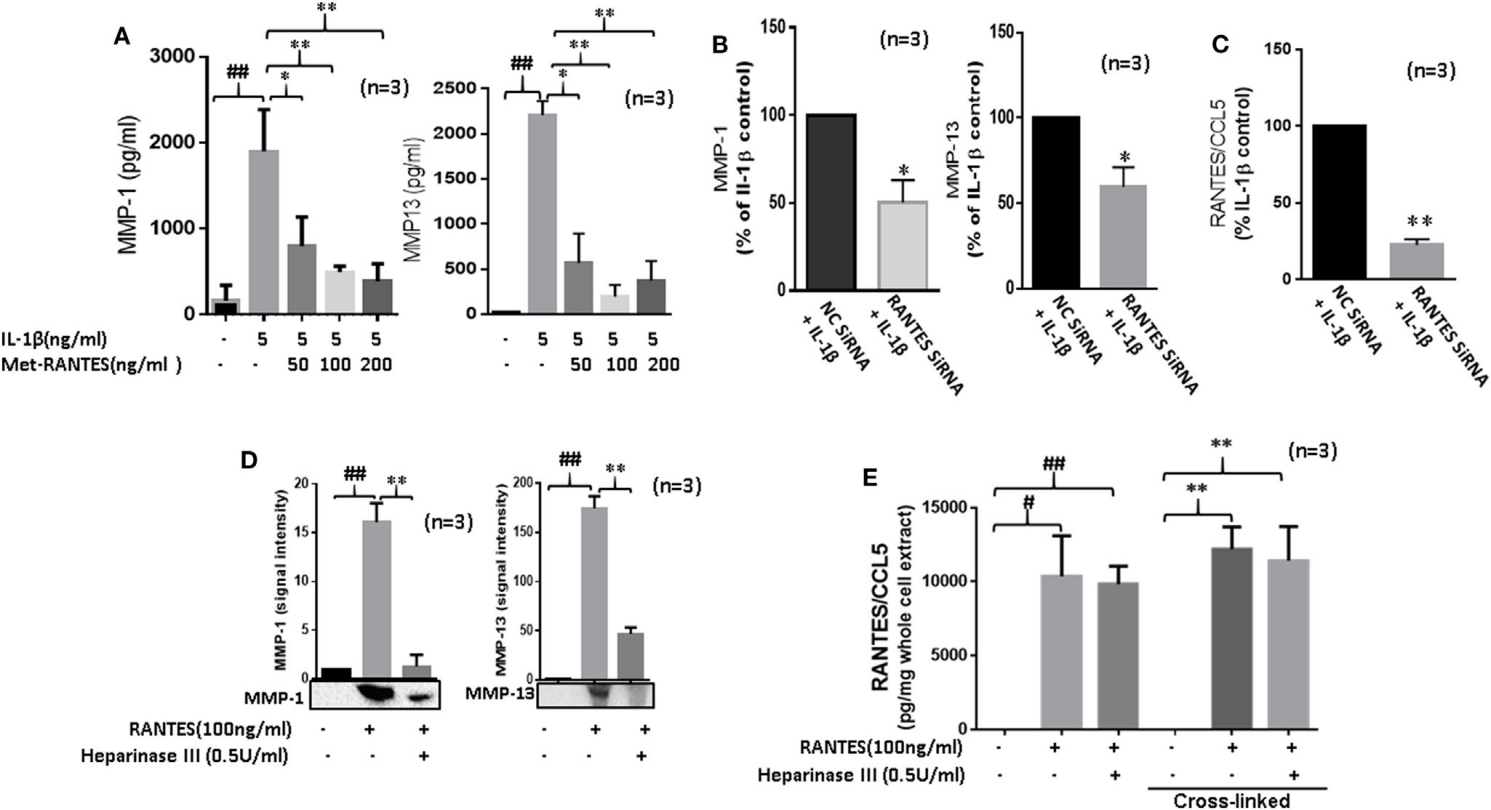
Regulated on activation, normal T expressed, and secreted (RANTES)/CC ligand 5 (CCL5) contributes to the proinflammatory cytokine-mediated matrix metalloproteinase (MMP)-1 and MMP-13 expression in rheumatoid arthritis (RA). **(A)** Met-RANTES inhibits interleukin (IL)-1β-induced MMP-1 and MMP-13 production in rheumatoid arthritis synovial fibroblasts (RASFs). **(B)** Effect of RANTES/CCL5 knockdown on IL-1β-induced MMP-1 and MMP-13 production in RASFs. RASFs were transfected with RANTES/CCL5-specific small-interfering RNA (siRNA) for 48 h and then stimulated with IL-1β (5 ng/ml) for 24 h. Levels of MMP-1 and MMP-13 in conditioned media was quantified using ELISA and represented as percent of IL-1β-treatment control. **(C)** Conditioned media from the above treated samples were also evaluated to determine percent knockdown of RANTES/CCL5 using siRNA approach and represented as percent of IL-1β treatment control. **(D)** Degradation of heparan sulfate proteoglycans (HSPG) by heparinase III reduces RANTES/CCL5-induced MMP-1 and MMP-13 expression in RASFs. RASFs were pretreated with heparinase III (0.5 U/ml) for 2 h followed by stimulation with RANTES/CCL5 (100 ng/ml) for 24 h. Conditioned media was concentrated and used to detect MMP-1 and MMP-13 expression using Western immunoblotting. Densitometry was performed to determine the relative changes. **(E)** RASFs pretreated with heparinase III (0.5 U/ml) for 2 h followed by stimulation with RANTES/CCL5 (100 ng/ml) for 1 h were used with or without cross-linking step for the detection of RANTES/CCL5 in the whole cell lysates (25 µl). Values are represented as mean ± SE from three independent experiments performed using RASFs from different donors under similar conditions. ##*p* < 0.01 for NS vs. or RANTES/CCL5 or IL-1β. *p < 0.05 or **p < 0.01 for RANTES/CCL5 or IL-1β vs. Met-RANTES or heparinase III treatment.

Interleukin-1β causes inflammation and tissue destruction in RA (1, 22). While IL-1β is a potent inducer of RANTES/CCL5 (22, 23), the contribution of RANTES/CCL5 in its tissue destructive property is not clear. Thus, we pretreated RASFs with Met-RANTES (50, 100, and 200 ng/ml) for 30 min, followed by IL-1β (5 ng/ml) stimulation for 24 h. Results showed that Met-RANTES markedly reduced the ability of IL-1β to produce MMP-1 (~79%) and MMP-13 (~64%) in RASFs (Figure 3A). Confirmation of these findings by siRNA approach showed that IL-1β-induced MMP-1 and MMP-13 production was indeed reduced by ~45% in RANTES/CCL5 siRNA-treated group when compared to their respective values in the negative control (NC) siRNA group (Figure 3B). We observed that siRNA targeting RANTES/CCL5 was capable of inhibiting IL-1β-induced RANTES/CCL5 production by almost 75% (Figure 3C).

### RANTES/CCL5 Requires HSPGs to Induce MMP-1 and MMP-13 Expression in RASFs

In addition to their critical role in developing chemokine gradient around activated endothelium, HSPGs cooperate with integrins and other cell adhesion receptors to facilitate cell-ECM attachment, cell–cell interactions, and cell motility (24). To determine their possible role in RANTES/CCL5-induced MMP-1 and MMP-13 production, RASFs were pretreated with heparinase III for 2 h followed by RANTES/CCL5 stimulation for 24 h. Analysis of the conditioned media showed that heparinase III significantly inhibits RANTES/CCL5-induced MMP-1 and MMP-13 expression (Figure 3D). To confirm whether heparinase III inhibits RANTES/CCL5 binding to the cell membrane receptor, we analyzed whole cell lysates from RASFs treated with RANTES/CCL5 (100 ng/ml) with or without heparinase III pretreatment for bound RANTES/CCL5 using ELISA kits. Our results showed that the levels of cell-membrane bound RANTES/CCL5 remained unaltered with heparinase III treatment (Figure 3E), suggesting that heparinase III may be able to block or interfere with RANTES/CCL5 signaling by a mechanism independent of RANTES/CCL5 binding.

### RANTES/CCL5 Selectively Activates PKCδ, JNK, and ERK Pathways in Human RASFs

**Figure 4:**
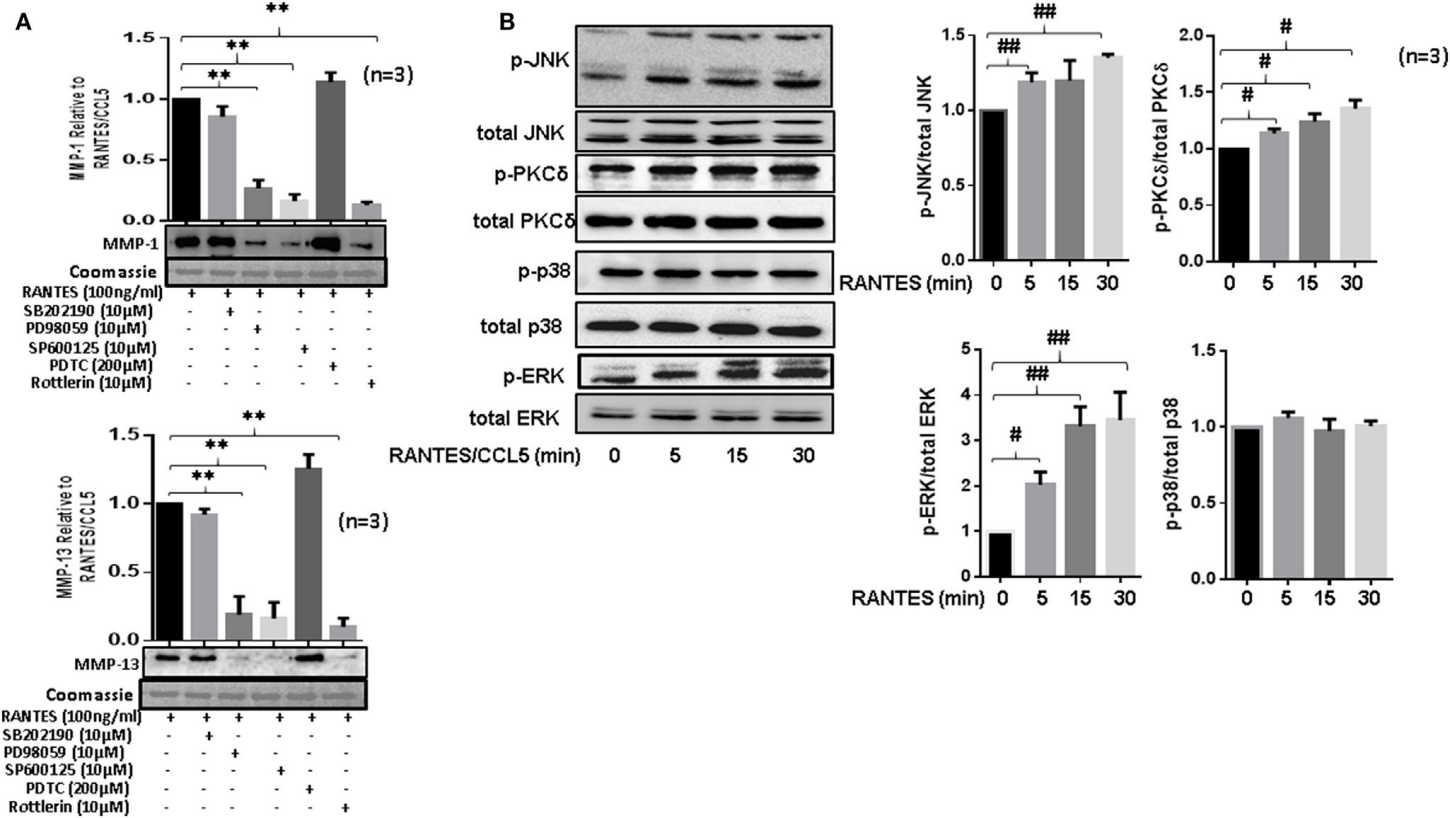
Regulated on activation, normal T expressed, and secreted (RANTES)/CC ligand 5 (CCL5) selectively activates PKCδ and JNK pathways to induce matrix metalloproteinase (MMP)-1 and MMP-13 production in rheumatoid arthritis synovial fibroblast (RASFs). **(A)** RASFs were pretreated with p-38 inhibitor (SB203980; 10 µM), ERK inhibitor (PD98059; 10 µM), JNK inhibitor (SP600125; 10 µM), NF-KB inhibitor (PDTC; 200 µM), or PKCδ inhibitor (Rottlerin; 10 µM) for 2 h followed by stimulation with RANTES/CCL5 for 24 h. Culture supernatant were concentrated to determine MMP-1 and MMP-13 expression using Western immunoblotting. Densitometry was performed to determine the relative changes. Gels were stained with Commassie Blue R250 stain to ensure equal loading. **(B)** Effect of RANTES/CCL5 (100 ng/ml) on the MAPK (p38, JNK and ERK) and PKCδ pathways was studied in human RASFs. RASFs were treated with RANTES (100 ng/ml) for different time (5, 15, and 30 min) and effect on phosphorylation of p38, JNK, ERK, and PKCδ was determined using Western immunoblotting. Total forms of p-38, JNK, ERK, and PKCδ were used as normalizing controls, respectively. Densitometry was performed to show the relative changes. Values are represented as mean ± SE from three or more independent experiments using cells from different donors under similar conditions. **p < 0.01 for RANTES/CCL5 vs. inhibitors; #*p* < 0.05 or ##*p* < 0.01 for NS vs. RANTES/CCL5.

To study the signaling pathways involved in RANTES/CCL5-induced MMP-1 and MMP-13 production, RASFs were pretreated with the inhibitor of p38 (SB202192), ERK (PD98059), JNK (SP600125), NF-κB (PDTC), or PKCδ (Rottlerin) for 2 h, followed by RANTES/CCL5 stimulation for 24 h. The conditioned media was concentrated and used to determine MMP-1 and MMP-13 production using Western immunoblotting. Our results showed that only the inhibition of PKCδ, JNK, or ERK markedly reduced RANTES/CCL5-induced MMP-1 and MMP-13 production in RASFs (Figure 4A). In light of these observations, we evaluated the effect of RANTES/CCL5 (100 ng/ml) on the activation of MAPK (p38, JNK, and ERK) and PKCδ pathways at different time points (5, 15, and 30 min). Western blot analysis of the cell lysates and further densitometric analysis showed that RANTES/CCL5 selectively activates p-PKCδ, p-JNK, and p-ERK in RASFs (Figure 4B).

### PKCδ Is an Important Upstream Mediator of RANTES/CCL5-Induced Signaling Pathways in RASFs

**Figure 5:**
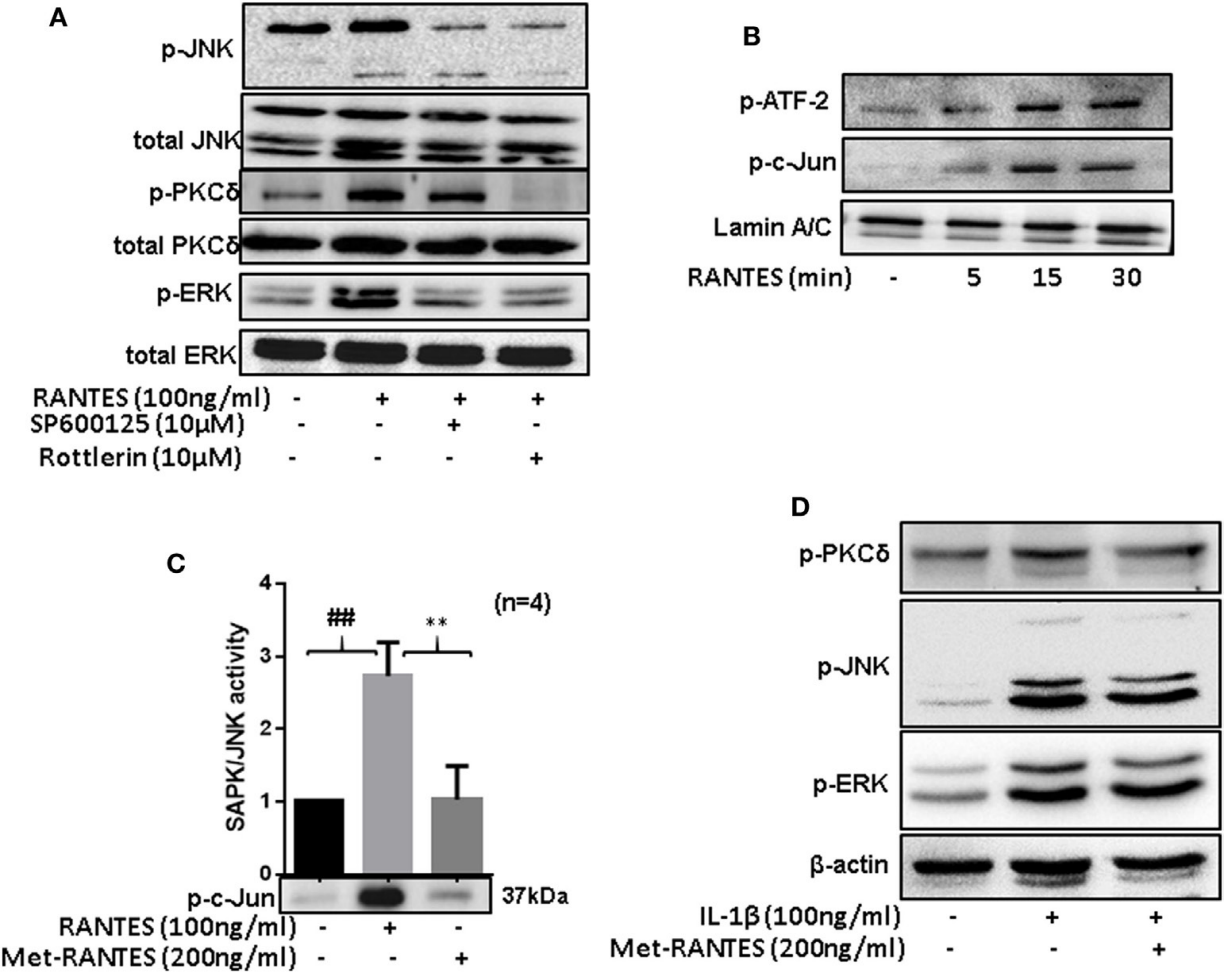
Regulated on activation, normal T expressed, and secreted (RANTES)/CC ligand 5 (CCL5)-mediated phosphorylation of PKCδ causes JNK and ERK activation to induce matrix metalloproteinase (MMP)-1 and MMP-13 production in rheumatoid arthritis synovial fibroblasts (RASFs). **(A)** RASFs were pretreated with the inhibitor of JNK (SP 600125; 10 µM) or PKCδ (Rottlerin; 10 µM) for 2 h followed by RANTES/CCL5 stimulation (100 ng/ml) for 30 min to determine the hierarchy of signaling proteins (p-PKCδ, p-JNK, and p-ERK) in RANTES/CCL5-mediated signal transduction using Western immunoblotting. **(B)** Effect of RANTES/CCL5 on nuclear translocation of p-ATF-2 and p-c-Jun in RASFs was determined using Western immunoblotting. RASFs were stimulated with RANTES/CCL5 (100 ng/ml) for 5, 15, and 30 min and nuclear fraction were used to study the effect on p-ATF-2 and p-c-Jun. Lamin A/C was used as loading control. **(C)** Effect of Met-RANTES on RANTES/CCL5 induced SAPK/JNK activity in RASFs. RASFs were pretreated with Met-RANTES for 30 min followed by RANTES/CCL5 treatment for 30 min. Immunoprecipitation was performed using p-SAPK/JNK antibody and antagonizing effect of Met-RANTES was determined by Western immunoblotting. Densitometry was performed to show the relative changes in SAPK/JNK activity and values are represented as mean ± SE from independent experiments performed on RASFs obtained from different donors. **(D)** RASFs were pretreated with Met-RANTES for 30 min followed by IL-1β treatment for 30 min. Cell lysates were used for the detection of p-PKCδ, p-JNK, and p-ERK. ##*p* < 0.01 NS vs. RANTES/CCL5; **p < 0.01 RANTES vs. RANTES + Met-RANTES.

To determine the hierarchy of signaling proteins in RANTES/CCL5 mediated activation of PKCδ and MAPK pathways, RASFs were pretreated with PKCδ inhibitor (Rottlerin) or JNK inhibitor (SP600125) for 2 h and then stimulated with RANTES/CCL5 (100 ng/ml) for 30 min. Cell lysates were evaluated for phosphorylation of JNK, ERK, and PKCδ. Rottlerin markedly suppressed RANTES/CCL5-induced phosphorylation of JNK, ERK, and PKCδ in RASFs (Figure 5A; Figure S2A in Supplementary Material). We also observed that the phosphorylation of PKCδ was partially affected by JNK inhibitor, suggesting PKCδ lies upstream of JNK/ERK in RANTES/CCL5-induced signaling cascade is RASFs (Figure 5A; Figure S2A in Supplementary Material).

Rheumatoid arthritis synovial fibroblasts were treated with RANTES/CCL5 for 5, 15, and 30 min and the expression of p-c-Jun and p-ATF-2 was determined in the nuclear extracts. Our results showed a time-dependent increase in the nuclear translocation of p-ATF-2 and p-c-Jun in RANTES/CCL5 stimulated RASFs (Figure 5B). Furthermore, we evaluated the effect of RANTES/CCL5 (100 ng/ml) alone or with Met-RANTES on SAPK/JNK *in vitro* kinase activity. Our results showed that RANTES/CCL5 significantly induced JNK kinase activity compared to the unstimulated control, which was completely inhibited by Met-RANTES pretreatment (Figure 5C).

To further understand the signaling mechanisms through which Met-RANTES was able to inhibit IL-1β-induced MMP-1 and MMP-13 production in RASFs (seen in Figure 3A), we pretreated RASFs with Met-RANTES (100 ng/ml) for 30 min, followed by IL-1β (10 ng/ml) stimulation for 30 min. Western blotting followed by the densitometric analysis of the cell lysates showed that Met-RANTES was able to inhibit p-JNK activation by roughly 30%, whereas it elicited modest inhibitory effects on p-PKCδ and p-ERK activation by IL-1β stimulation (Figure 5D; Figure S2B in Supplementary Material). These findings suggest that Met-RANTES inhibits IL-1β-induced MMP-1 and MMP-13 production by directly inhibiting JNK pathway in human RASFs *in vitro*.

### RANTES/CCL5 Induces Collagenolytic Activity in RA

**Figure 6:**
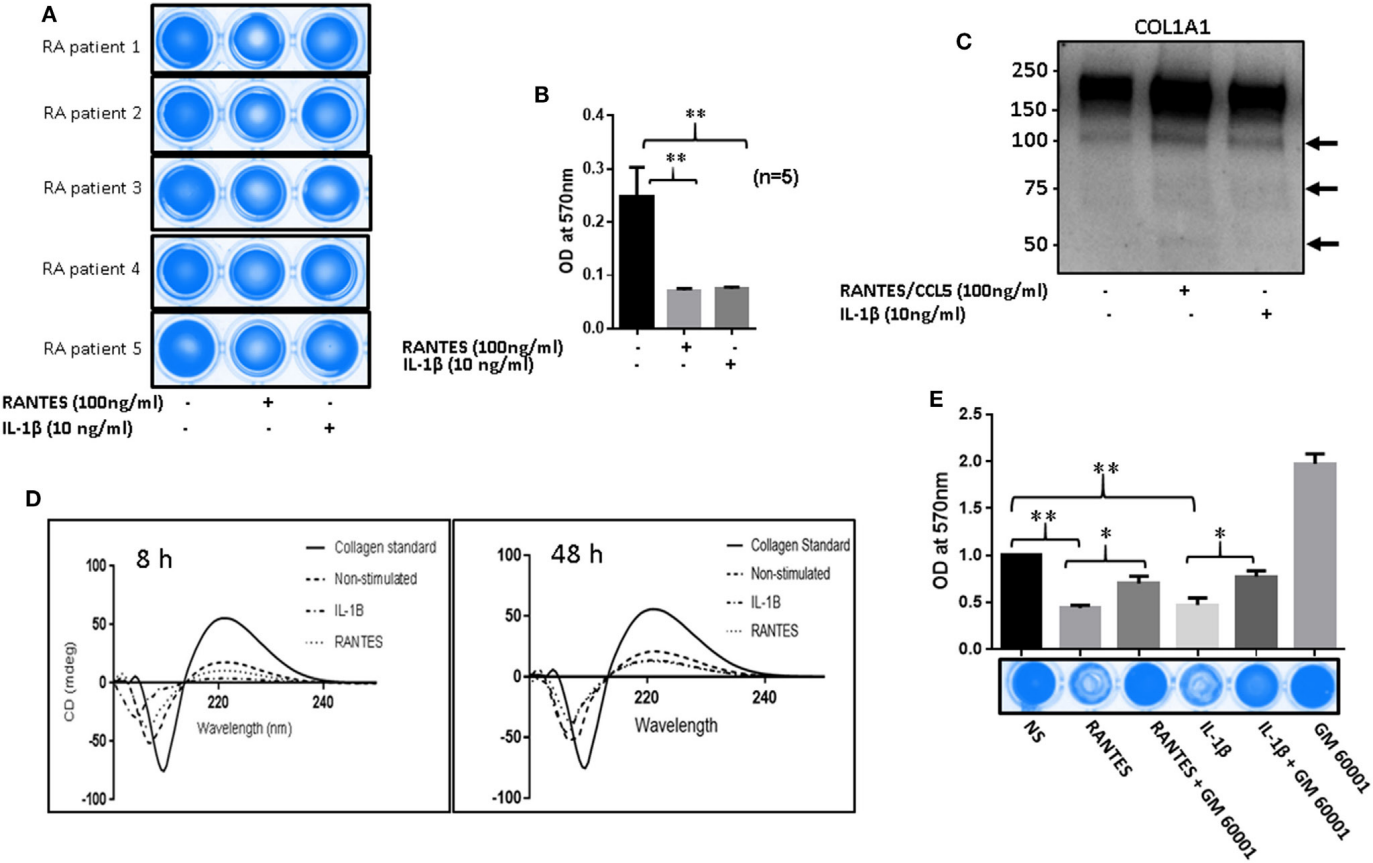
Regulated on activation, normal T expressed, and secreted (RANTES)/CC ligand 5 (CCL5) facilitates matrix metalloproteinases (MMPs)-mediated collagen degradation in rheumatoid arthritis (RA). **(A)** RA synovial fibroblasts (RASFs) from five donors were stimulated with RANTES/CCL5 (100 ng/ml) and IL-1β (10 ng/ml) for 24 h. Condition media was placed onto a 96-well plate coated with type I collagen film and incubated for 24 h. Plates were stained with Coomassie R250 analyzed by ChemiDoc™ Imager. **(B)** Optical density (570 nm) of the RANTES/CCL5 and IL-1β-stimulated samples compared to control is shown. **(C)** Condition media from the same treatment was concentrated using Amicon 10K ultra 0.5 centrifugal filters and analyzed for type I collagen by Western blotting. **(D)** Condition media from RANTES/CCL-5 or IL-1β stimulated RASFs, was applied on collagen coated 96-well plates for 8 and 48 h. Digested collagen was collected and CD spectroscopy was performed using a Jasco J-815 spectropolarimeter. **(E)** RASFs were pretreated with GM6001 (20 µM) for 2 h and then stimulated with RANTES/CCL5 (100 ng/ml) or IL-1β (10 ng/ml). Collagen type I plates were exposed for 24 h, media aspirated, and then stained with Coomassie R250 and analyzed as described in Section “Materials and Methods.” Densitometry was performed to show the relative changes. Values are represented as MEAN ± SE from experiments performed using cells from four to five donors. *p < 0.05 or **p < 0.01.

To determine the collagenolytic potential of RANTES/CCL5, RASFs were stimulated with RANTES/CCL5 (100 ng/ml) or IL-1β (10 ng/ml) for 24 h. Condition media (200 µl) was added to type I collagen coated 96-well plates and incubated for 24 h. At the time of termination, media was collected and plates were stained with Coomassie blue R250. Our results showed that collagen was digested and eroded from the wells exposed to RANTES/CCL5 or IL-1β-treated conditioned media compared to unstimulated control, indicating their collagenolytic potentials (Figure 6A). When these stained plates were further analyzed for absorbance at 570 nm, both RANTES/CCL5 and IL-1β-treated RASFs showed significant loss of collagen further confirming the ability of RANTES/CCL5 and IL-1β to induce collagen degradation by MMP-1 and MMP-13 production (Figure 6B). Western blot evaluation of the conditioned media for the digested/released collagen fragments from the coated plates for COL1A1 expression showed a marked increase in the collagen fragments, in addition to the native 220 kDa, generated in both RANTES/CCL5 and IL-1β stimulated samples (Figure 6C).

To further confirm collagen degradation, the conditioned media from similar experiments (8 h as early and 48 h as a late time point) were evaluated for structural changes in collagen and compared to the native collagen standard using CD spectroscopy (Figure 6D). Compared to the native collagen, exposure to unstimulated conditioned media, obtained from RA-affected samples, showed a marked change in the structure of collagen (Figure 6D). In addition, RANTES/CCL5 or IL-1β-treated conditioned media showed further conformational changes to collagen structure as evident by the loss of the characteristic collagen peaks at 200 (negative) and 225 (positive) nm (21), suggesting collagen degradation (Figure 6D). We also compared the untreated conditioned media from NLSFs with untreated RASFs to identify that while there were modest structural changes brought by RASF’s compared to NLSF’s untreated media (Figure S3 in Supplementary Material). These findings suggest that chronic activation of RASFs by RANTES/CCL5 or cytokines such as IL-1β during the disease progression leads to the subtle structural changes in the native collagen of the cartilage that eventually contribute to the tissue destruction in RA.

To verify whether this damage to collagen by RANTES/CCL5 or IL-1β were mediated by MMPs, RASFs were pretreated with a broad-spectrum MMP inhibitor (GM6001; 20 µM) for 2 h and then stimulated with RANTES/CCL5 or IL-1β for 24 h. The conditioned media was added on to type I collagen-coated plates as described above. A significantly higher degradation of type I collagen was found in both RANTES/CCL5 or IL-1β alone treated as evidenced by the reduced Coomassie staining (Figure 6E). However, pretreatment with GM6001 showed a marked inhibition of collagen degradation in RANTES/CCL5 or IL-1β-treated group (Figure 6E). These results suggest that the effect of both RANTES/CCL5 and IL-1β on type I collagen degradation are orchestrated by MMP-1 and MMP-13 production in RASFs.

## Discussion

This study provides a novel mechanistic insight to the role of RANTES/CCL5 in inducing MMP-1 and MMP-13 expression in human RASFs and its damaging impact on the structure of native collagen. Furthermore, the study also showed that IL-1β-induced MMP-1 and MMP-13 is partly mediated *via* RANTES/CCL5 and there exists an opportunity to therapeutically limit the role of RANTES/CCL5 in the process of tissue destruction in RA by reducing its interaction with HSPGs found on the cell surface and ECM, or inhibiting the signaling proteins such as PKCδ in RASFs.

Regulated on activation, normal T expressed, and secreted (RANTES)/CCL5 is produced by the majority of cell types that participate in pathogenesis of RA, including SFs, endothelial cells, chondrocytes, monocyte/macrophages, and activated T cells (11, 12). RANTES/CCL5 among other CC chemokines is an established regulator of migration, cell proliferation, and leukocyte trafficking. In our previous studies, we have shown that IL-1β is a potent inducer of RANTES/CCL5 and other chemokines in human RASFs (1, 23). Administration of Met-RANTES has been shown to ameliorate experimental arthritis by reducing joint inflammation and bone destruction (25, 26). Our results showing that RANTES/CCL5 is capable of inducing MMP-1 and MMP-13 expression and collagenase activity and may also contribute to IL-1β-induced destruction further implicates RANTES/CCL5 as a potent mediator of bone and cartilage damage in RA. Whether RANTES/CCL5 acts together, in a synergistic manner, with IL-1β to exacerbate inflammation and tissue destruction by RASFs require further experiments.

An extensive joint destruction in RA, as well as OA, is mediated by an intense action of various proteinases, the most important among them being collagenases (14, 27). MMP-1 and MMP-13 are produced by RASFs and their elevated levels in the synovial fluid and enhanced expression in synovial tissue biopsies from RA patients provide an evidence for their active role in tissue destruction (28, 29). Firestein et al. have demonstrated that JNK pathway mediates cytokine-induced MMP-1 and MMP-13 expression in SFs and murine inflammatory arthritis model (30, 31). The promoter region of MMP-1 and MMP-13 hosts several AP-1-binding sites, which makes it an important transcriptional regulator of these MMPs (32). In addition, IL-1β has been shown to be a more potent inducer of MMP-1 compared to TNF-α partly due to its ability to induce ERK pathway concomitantly (33), suggesting that ERK may play a supportive role in the transcriptional activation of MMP-1, and possibly MMP-13. In fact, the regulation by PKCδ of its downstream signaling proteins JNK/ERK and the inhibition of RANTES/CCL5-induced MMP-1 and MMP-13 expression by Rottlerin suggest that PKCδ might be a relevant therapeutic target in RA. This is further supported by a previous study, in which RANTES/CCL5 induced IL-6 production in human OASFs *via* PKCδ and Src pathways (27). Recent clinical studies using JAK inhibitor (tofacitinib) or anti-TNF therapy have also shown efficacy in reducing tissue destruction, in part by downregulating MMP-1 and MMP-13 expression (34, 35).

We observed the inhibition of IL-1β-induced MMP-1 and MMP-13 expression by Met-RANTES, which may partly be due to its ability to inhibit the activation of JNK pathway. While these findings are open to different interpretations, we hypothesize that Met-RANTES is effective in blocking IL-1β in a two-pronged approach: by blocking the contribution of RANTES/CCL5 produced in response to IL-1β stimulation of RASFs and by inhibiting the phosphorylation and kinase activity of JNK, thereby producing a profound decrease in MMP-1 and MMP-13 production. Our previous findings have shown PKCδ as one of the key molecules in IL-1β signaling pathway (1, 23), however, other studies suggest that the regulation of JNK activation that eventually reduces AP-1 nuclear translocation and DNA-binding activity may exhibit significant inhibition of MMP activity in RASFs (30, 31, 33). Further detailed studies are warranted to confirm the effect of Met-RANTES or any other commercially available structural anatagonists of RANTES/CCL5 on cytokine signaling network in RA pathogenesis.

Heparan sulfate proteoglycans not only protect cytokines and chemokines from proteolysis, they also facilitate the formation of chemokine gradients involved in leukocyte recruitment and homing (36). Recent study showed that the selective induction of a CXCL8-binding site on endothelial syndecan-3, a transmembrane HSPG, is increased in RA synovium, which may be involved in leukocyte trafficking into RA synovial tissue (37). The syndecan family of transmembrane proteoglycans are the major source of cell surface HS in all cell types and provides complex role in inflammation (38, 39). The inhibition of RANTES/CCL5-induced MMP-1 and MMP-13 production by the depletion of HSPGs using heparinase III in RASF cultures, further confirms that in addition to CC receptor-mediated signaling, RANTES/CCL5 also utilizes HSPGs to activate signal transduction pathways in RASFs. However, our results showed that heparinase III has no effect on RANTES/CCL5 binding to the cell surface, which suggests that RANTES/CCL5 utilizes other proteoglycans present on the cell surface to bind and activate its signaling. Since RASFs are a major source of RANTES/CCL5 in the affected joints, there is a possibility that it may not rely on HSPGs to activate its signal transduction pathways. On the contrary, it can also be postulated that besides HSPGs, heparinase III may also affect other cell surface receptors by proteolytic shedding or structural modification that impairs their signaling and downstream catabolic events. These findings further warrant more elaborate study for a better therapeutic understanding of heparinase III and MMP axis. The effector molecules capable of degrading type I and II collagen within the triple helix structure are known to be attributed largely to MMPs and cysteine proteases (38). It is also known that human RASFs invade and degrade collagen rich structures associated with joints, including bone, cartilage, tendons, and ligaments (39). Here, we demonstrated that RASFs, when stimulated with RANTES/CCL5 express MMP-1 and MMP-13 that have profound collagenase activity to directly degrade cross-linked collagen networks.

In conclusion, our results from this study suggest that RANTES/CCL5 plays a role of an active mediator, not a passive bystander, in RA pathogenesis by activating RASFs to promote MMP-1 and MMP-13 mediated ECM destruction. While the therapeutic strategies aimed at exclusively targeting RANTES/CCL5 or CCR5 were not successful in RA, these findings provide a rationale for testing HSPGs or signaling proteins as an adjunct therapeutic target to limit RASF participation in disease pathogenesis.

## Author Contributions

SA, NA, and SA designed this study. SA and NA performed experiments and wrote the manuscript. SA participated in writing the manuscript and provided his support to the study. JW participated in writing the manuscript and conducted and analyzed CD spectroscopy experiments. SA is the corresponding author of the manuscript.

## Conflict of Interest Statement

The authors declare that the research was conducted in the absence of any commercial or financial relationships that could be construed as a potential conflict of interest.

## References

[1] Ahmed S Pakozdi A Koch AE. Regulation of interleukin-1beta-induced chemokine production and matrix metalloproteinase 2 activation by epigallocatechin-3-gallate in rheumatoid arthritis synovial fibroblasts. Arthritis Rheum (2006) 54(8):2393–401. 10.1002/art.22023 16869002

[2] Koch AE Chemokines and their receptors in rheumatoid arthritis: future targets? Arthritis Rheum (2005) 52(3):710–21. 10.1002/art.20932 15751074

[3] Katschke KJ Jr Rottman JB Ruth JH Qin S Wu L LaRosa G Differential expression of chemokine receptors on peripheral blood, synovial fluid, and synovial tissue monocytes/macrophages in rheumatoid arthritis. Arthritis Rheum (2001) 44(5):1022–32. 10.1002/1529-0131(200105)44:5<1022:AID-ANR181>3.0.CO;2-N 11352233

[4] Stievano L Piovan E Amadori A. C and CX3C chemokines: cell sources and physiopathological implications. Crit Rev Immunol (2004) 24(3):205–28. 10.1615/CritRevImmunol.v24.i3.40 15482255

[5] Zlotnik A Yoshie O Chemokines: a new classification system and their role in immunity. Immunity (2000) 12(2):121–7. 10.1016/S1074-7613(00)80165-X 10714678

[6] Jones BA Riegsecker S Rahman A Beamer M Aboualaiwi W Khuder SA Role of ADAM-17, p38 MAPK, cathepsins, and the proteasome pathway in the synthesis and shedding of fractalkine/CX(3) CL1 in rheumatoid arthritis. Arthritis Rheum (2013) 65(11):2814–25. 10.1002/art.38095 23897050

[7] Ludwig A Weber C. Transmembrane chemokines: versatile ‘special agents’ in vascular inflammation. Thromb Haemost (2007) 97(5):694–703. 10.1160/TH07-01-0035 17479179

[8] Mor A Abramson SB Pillinger MH. The fibroblast-like synovial cell in rheumatoid arthritis: a key player in inflammation and joint destruction. Clin Immunol (2005) 115(2):118–28. 10.1016/j.clim.2004.12.009 15885632

[9] Patel DD Zachariah JP Whichard LP. CXCR3 and CCR5 ligands in rheumatoid arthritis synovium. Clin Immunol (2001) 98(1):39–45. 10.1006/clim.2000.4957 11141325

[10] Hosaka S Akahoshi T Wada C Kondo H. Expression of the chemokine superfamily in rheumatoid arthritis. Clin Exp Immunol (1994) 97(3):451–7. 10.1111/j.1365-2249.1994.tb06109.x 7521808 PMC1534851

[11] Iwamoto T Okamoto H Toyama Y Momohara S. Molecular aspects of rheumatoid arthritis: chemokines in the joints of patients. FEBS J (2008) 275(18):4448–55. 10.1111/j.1742-4658.2008.06580.x 18662305

[12] Ahmed S Rahman A Hasnain A Goldberg VM Haqqi TM. Phenyl N-tert-butylnitrone down-regulates interleukin-1 beta-stimulated matrix metalloproteinase-13 gene expression in human chondrocytes: suppression of c-Jun NH2-terminal kinase, p38-mitogen-activated protein kinase and activating protein-1. J Pharmacol Exp Ther (2003) 305(3):981–8. 10.1124/jpet.102.048611 12626640

[13] Ishiguro N Ito T Oguchi T Kojima T Iwata H Ionescu M Relationships of matrix metalloproteinases and their inhibitors to cartilage proteoglycan and collagen turnover and inflammation as revealed by analyses of synovial fluids from patients with rheumatoid arthritis. Arthritis Rheum (2001) 44(11):2503–11. 10.1002/1529-0131(200111)44:11<2503::AID-ART430>3.0.CO;2-P 11710706

[14] Eck SM Blackburn JS Schmucker AC Burrage PS Brinckerhoff CE. Matrix metalloproteinase and G protein coupled receptors: co-conspirators in the pathogenesis of autoimmune disease and cancer. J Autoimmun (2009) 33(3–4):214–21. 10.1016/j.jaut.2009.09.011 19800199 PMC2783549

[15] Burrage PS Mix KS Brinckerhoff CE. Matrix metalloproteinases: role in arthritis. Front Biosci (2006) 11:529–43. 10.2741/1817 16146751

[16] Singh AK Umar S Riegsecker S Chourasia M Ahmed S Regulation of transforming growth factor beta-activated kinase activation by epigallocatechin-3-gallate in rheumatoid arthritis synovial fibroblasts: suppression of K(63)-linked autoubiquitination of tumor necrosis factor receptor-associated factor 6. Arthritis Rheumatol (2016) 68(2):347–58. 10.1002/art.39447 26473505 PMC5383419

[17] Akhtar N Singh AK Ahmed S MicroRNA-17 suppresses TNF-alpha signaling by interfering with TRAF2 and cIAP2 association in rheumatoid arthritis synovial fibroblasts. J Immunol (2016) 197(6):2219–28. 10.4049/jimmunol.1600360 27534557 PMC5010933

[18] Kiener HP Watts GF Cui Y Wright J Thornhill TS Skold M Synovial fibroblasts self-direct multicellular lining architecture and synthetic function in three-dimensional organ culture. Arthritis Rheum (2010) 62(3):742–52. 10.1002/art.27285 20131230

[19] Ahmed S Marotte H Kwan K Ruth JH Campbell PL Rabquer BJ Epigallocatechin-3-gallate inhibits IL-6 synthesis and suppresses transsignaling by enhancing soluble gp130 production. Proc Natl Acad Sci U S A (2008) 105(38):14692–7. 10.1073/pnas.0802675105 18796608 PMC2567200

[20] Sabeh F Fox D Weiss SJ. Membrane-type I matrix metalloproteinase-dependent regulation of rheumatoid arthritis synoviocyte function. J Immunol (2010) 184(11):6396–406. 10.4049/jimmunol.0904068 20483788

[21] Jariashvili K Madhan B Brodsky B Kuchava A Namicheishvili L Metreveli N. UV damage of collagen: insights from model collagen peptides. Biopolymers (2012) 97(3):189–98. 10.1002/bip.21725 22002434 PMC3299808

[22] Noh EM Kim JS Hur H Park BH Song EK Han MK Cordycepin inhibits IL-1beta-induced MMP-1 and MMP-3 expression in rheumatoid arthritis synovial fibroblasts. Rheumatology (2009) 48(1):45–8. 10.1093/rheumatology/ken417 19056796

[23] Marotte H Ruth JH Campbell PL Koch AE Ahmed S. Green tea extract inhibits chemokine production, but up-regulates chemokine receptor expression, in rheumatoid arthritis synovial fibroblasts and rat adjuvant-induced arthritis. Rheumatology (2010) 49(3):467–79. 10.1093/rheumatology/kep397 20032224 PMC2820264

[24] Couchman JR Syndecans: proteoglycan regulators of cell-surface microdomains? Nat Rev Mol Cell Biol (2003) 4(12):926–37. 10.1038/nrm1257 14685171

[25] Shahrara S Proudfoot AE Woods JM Ruth JH Amin MA Park CC Amelioration of rat adjuvant-induced arthritis by Met-RANTES. Arthritis Rheum (2005) 52(6):1907–19. 10.1002/art.21033 15934086 PMC1282452

[26] Plater-Zyberk C Hoogewerf AJ Proudfoot AE Power CA Wells TN. Effect of a CC chemokine receptor antagonist on collagen induced arthritis in DBA/1 mice. Immunol Lett (1997) 57(1–3):117–20. 10.1016/S0165-2478(97)00075-8 9232436

[27] Tang CH Hsu CJ Fong YC. The CCL5/CCR5 axis promotes interleukin-6 production in human synovial fibroblasts. Arthritis Rheum (2010) 36:3615–24. 10.1002/art.27755 20862675

[28] Yoshihara Y Nakamura H Obata K Yamada H Hayakawa T Fujikawa K Matrix metalloproteinases and tissue inhibitors of metalloproteinases in synovial fluids from patients with rheumatoid arthritis or osteoarthritis. Ann Rheum Dis (2000) 59(6):455–61. 10.1136/ard.59.6.455 10834863 PMC1753174

[29] Miller MC Manning HB Jain A Troeberg L Dudhia J Essex D Membrane type 1 matrix metalloproteinase is a crucial promoter of synovial invasion in human rheumatoid arthritis. Arthritis Rheum (2009) 60(3):686–97. 10.1002/art.24331 19248098 PMC2819053

[30] Han Z Boyle DL Aupperle KR Bennett B Manning AM Firestein GS Jun N-terminal kinase in rheumatoid arthritis. J Pharmacol Exp Ther (1999) 291(1):124–30. 10490895

[31] Han Z Boyle DL Chang L Bennett B Karin M Yang L c-Jun N-terminal kinase is required for metalloproteinase expression and joint destruction in inflammatory arthritis. J Clin Invest (2001) 108(1):73–81. 10.1172/JCI12466 11435459 PMC209341

[32] Vincenti MP Brinckerhoff CE. Transcriptional regulation of collagenase (MMP-1, MMP-13) genes in arthritis: integration of complex signaling pathways for the recruitment of gene-specific transcription factors. Arthritis Res (2002) 4(3):157–64. 10.1186/ar401 12010565 PMC128926

[33] Barchowsky A Frleta D Vincenti MP. Integration of the NF-kappaB and mitogen-activated protein kinase/AP-1 pathways at the collagenase-1 promoter: divergence of IL-1 and TNF-dependent signal transduction in rabbit primary synovial fibroblasts. Cytokine (2000) 12(10):1469–79. 10.1006/cyto.2000.0743 11023661

[34] Boyle DL Soma K Hodge J Kavanaugh A Mandel D Mease P The JAK inhibitor tofacitinib suppresses synovial JAK1-STAT signalling in rheumatoid arthritis. Ann Rheum Dis (2015) 74(6):1311–6. 10.1136/annrheumdis-2014-206028 25398374 PMC4431345

[35] Catrina AI Lampa J Ernestam S af Klint E Bratt J Klareskog L Anti-tumour necrosis factor (TNF)-alpha therapy (etanercept) down-regulates serum matrix metalloproteinase (MMP)-3 and MMP-1 in rheumatoid arthritis. Rheumatology (2002) 41(5):484–9. 10.1093/rheumatology/41.5.484 12011369

[36] Sarrazin S Lamanna WC Esko JD Heparan sulfate proteoglycans. Cold Spring Harb Perspect Biol (2011) 3(7):a004952 10.1101/cshperspect.a004952 21690215 PMC3119907

[37] Patterson AM Gardner L Shaw J David G Loreau E Aguilar L Induction of a CXCL8 binding site on endothelial syndecan-3 in rheumatoid synovium. Arthritis Rheum (2005) 52(8):2331–42. 10.1002/art.21222 16052590

[38] Bernfield M Gotte M Park PW Reizes O Fitzgerald ML Lincecum J Functions of cell surface heparan sulfate proteoglycans. Annu Rev Biochem (1999) 68:729–77. 10.1146/annurev.biochem.68.1.729 10872465

[39] Severin IC Soares A Hantson J Teixeira M Sachs D Valognes D Glycosaminoglycan analogs as a novel anti-inflammatory strategy. Front Immunol (2012) 3:293. 10.3389/fimmu.2012.00293 23087686 PMC3472544

