# Supplementary material for: RANTES/CCL5 Induces Collagen Degradation by Activating MMP-1 and MMP-13 Expression in Human Rheumatoid Arthritis Synovial Fibroblasts
Source: Front Immunol. 2017 Oct 18;8:1341. doi: 10.3389/fimmu.2017.01341 (PMC5651228; doi:10.3389/fimmu.2017.01341)
Supplement: Supplementary file 1 [file Image_1.pdf]

Supplementary Fig. 1

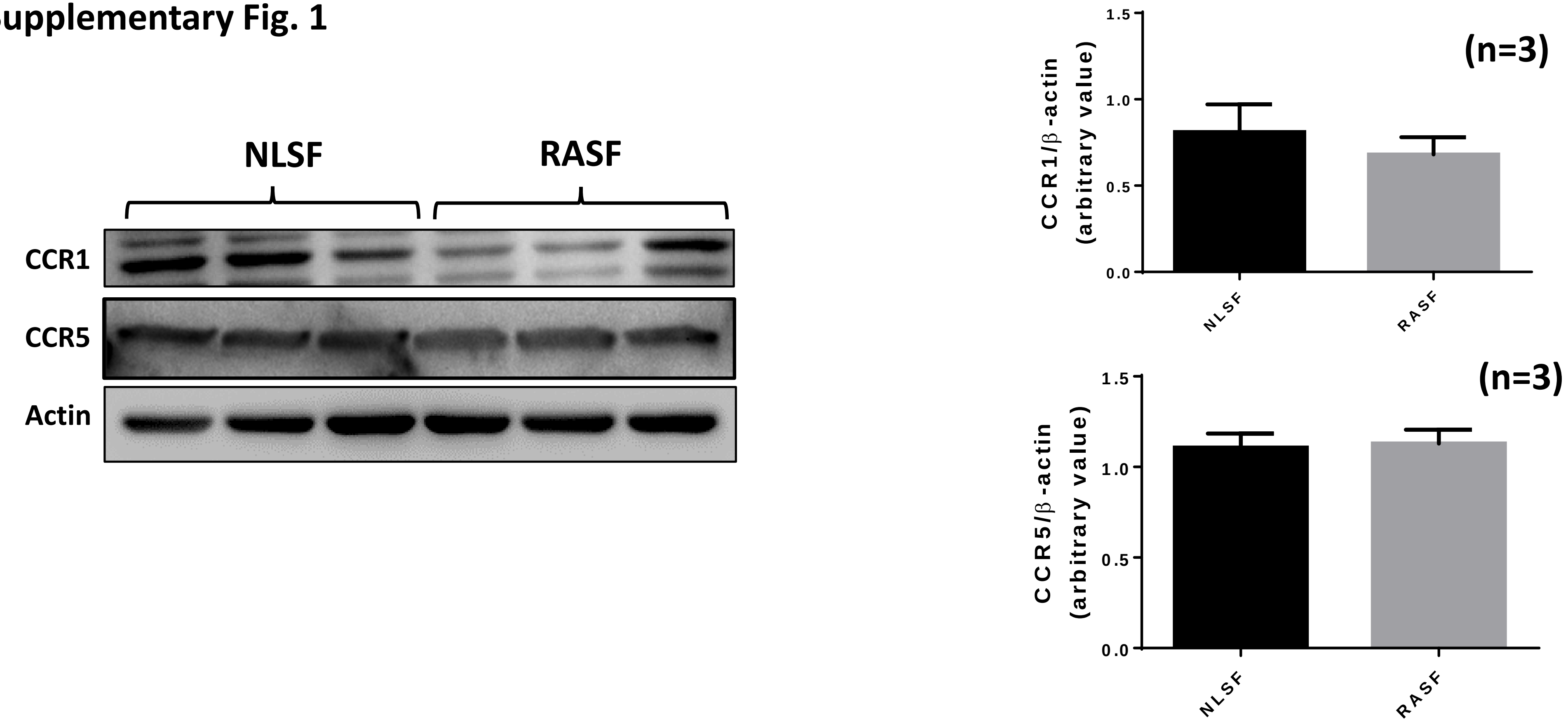

Fig. S1: RASFs exhibit low CCR1, bot not CCR5, expression compared to NLSFs. SFs from NL and RA donors were grown >85% confluent, serum-starved overnight and lysed to prepare whole cell extract (WCE). Equal amount of WCE (25 µg) from each sample was used in Western blot analysis to determine the expression of CCR1 and CCR5. Densitometric analysis was performed for each blot and the values were normalized with β-actin.

Supplementary Fig. 2

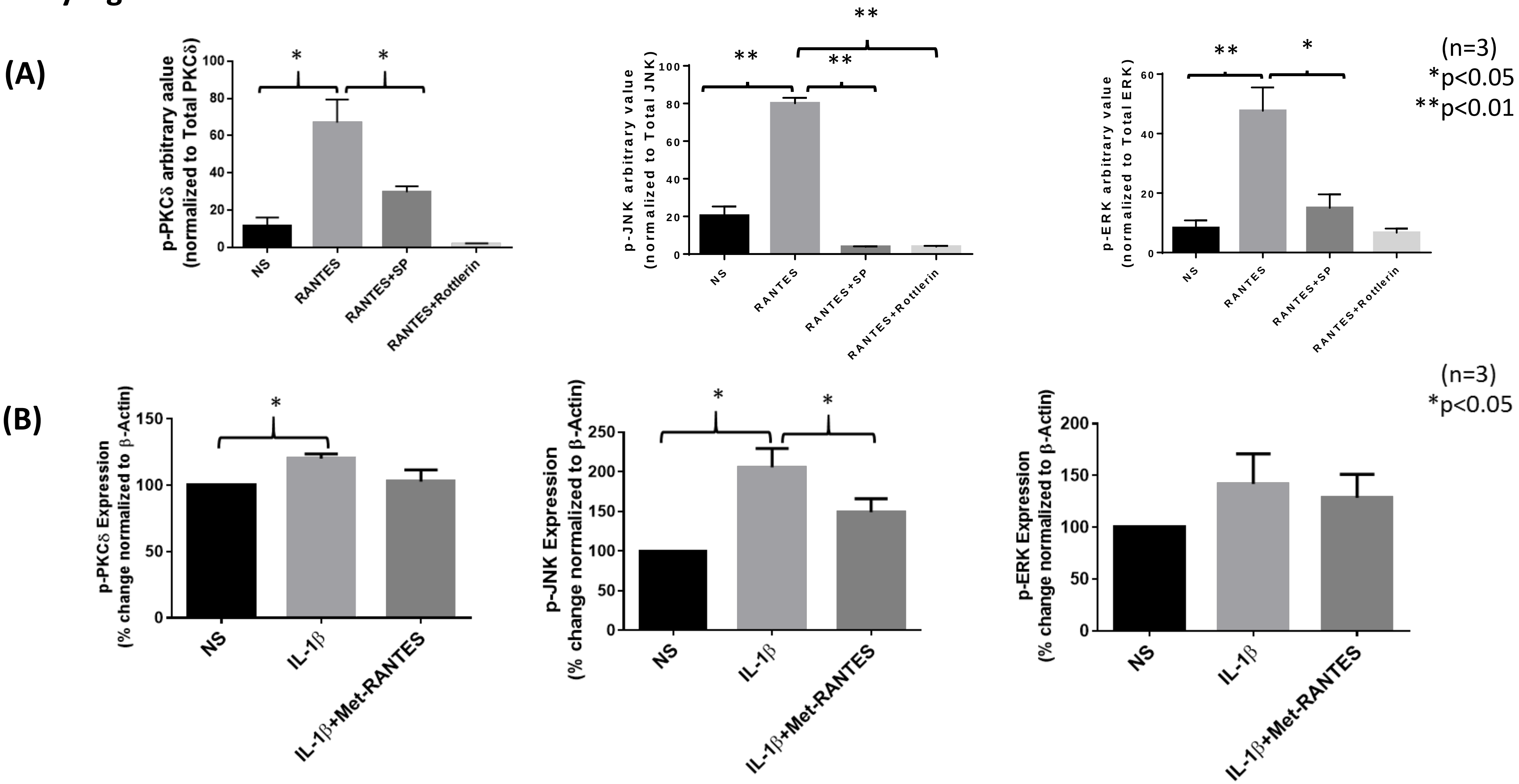

Fig. S2A&B: Densitometric analysis of Western blots analyzed for the activated signaling proteins. A, Densitometric analysis of the Western blots on the samples treated with RANTES/CCL5 alone or in the presence of a chemical inhibitor of JNK (SP600125) or PKCδ (Rottlerin) for p-PKCδ, p-JNK, and p-ERK expression. Values represent mean ± SEM of results obtained from three independent donor RASFs. **B,** RASFs were pretreated with Met-RANTES (100 ng/ml) for 30 min followed by IL-1β (10 ng/ml) for 30 min. Densitometric analysis of p-PKCδ, p-JNK, and p-ERK blots was performed and the values from three independent donor RASFs were presented as mean ± SEM. *p<0.05 and **p<0.01.

Supplementary Fig. 3

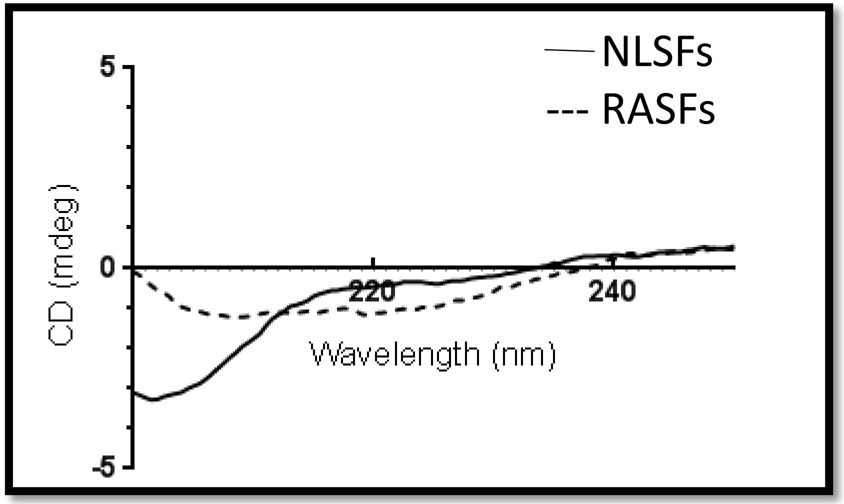

Fig. S3: CD Spectra suggests an inherent change in native collagen structure upon exposure to RASF's conditioned media. SFs from NL and RA donors were cultured to >85 confluence, serum-starved overnight and the conditioned media was collected. Two hundred microliters of each sample was added to each well in 96-well plate coated with type I collagen and experiment was performed as described in detail in "Materials and Methods" section. CD spectra analysis on the collagen samples collected from the wells exposed to NLSF's and RASF's untreated conditioned media showed a marked structural change in the RASF exposed collagen. The graph is a representative CD spectra of assay performed in triplicate.
